# Formononetin Activates the Nrf2/ARE Signaling Pathway Via Sirt1 to Improve Diabetic Renal Fibrosis

**DOI:** 10.3389/fphar.2020.616378

**Published:** 2021-01-13

**Authors:** Kai Zhuang, Xiyu Jiang, Renbin Liu, Cunsi Ye, Yumei Wang, Yunhan Wang, Shijian Quan, Heqing Huang

**Affiliations:** ^1^ School of Pharmaceutical Sciences, Guangzhou University of Chinese Medicine, Guangzhou, China; ^2^ Department of Traditional Chinese Medicine, Renmin Hospital, Hubei University of Medicine, Shiyan, China; ^3^ Science and Technology Innovation Center, Guangzhou University of Chinese Medicine, Guangzhou, China; ^4^ Laboratory of Pharmacology and Toxicology, School of Pharmaceutical Science, Sun Yat-Sen University, Guangzhou, China

**Keywords:** formononetin, diabetic nephropathy, renal fibrosis, oxidative stress, Sirt1/Nrf2/ARE signaling pathways

## Abstract

Oxidative stress is the main factor responsible for the induction of diabetic renal fibrosis. Thus, improving the state of oxidative stress can effectively prevent the further deterioration of diabetic nephropathy (DN). Previous research has shown that formononetin (FMN), a flavonoid with significant antioxidant activity and Sirt1 activation effect, can improve diabetic renal fibrosis. However, the exact mechanisms underlying the effect of FMN on diabetic renal fibrosis have yet to be elucidated. In this study, we carried out *in vivo* experiments in a db/db (diabetic) mouse model and demonstrated that FMN activated the nuclear factor E2-related factor 2 (Nrf2)/antioxidant response element (ARE) signaling pathway and improved oxidative stress by increasing levels of sirtuin-1 (Sirt1) protein level in renal tissue. We also found that this process reversed the up-regulation of fibronectin (FN) and intercellular adhesion molecule 1 (ICAM-1) and led to an improvement in renal insufficiency. *In vitro* results further showed that FMN significantly reversed the upregulation of FN and ICAM-1 in glomerular mesangial cells (GMCs) exposed to high glucose. FMN also promoted the expression of Nrf2 and widened its nuclear distribution. Thus, our data indicated that FMN inhibited hyperglycemia-induced superoxide overproduction by activating the Nrf2/ARE signaling pathway. We also found that FMN up-regulated the expression of Sirt1 and that Sirt1 deficiency could block the activation of the Nrf2/ARE signaling pathway in GMCs induced by high glucose. Finally, we found that Sirt1 deficiency could reverse the down-regulation of FN and ICAM-1 induced by FMN. Collectively, our data demonstrated that FMN up-regulated the expression of Sirt1 to activate the Nrf2/ARE signaling pathway, improved oxidative stress in DN to prevent the progression of renal fibrosis. Therefore, FMN probably represents an efficient therapeutic option of patients with DN.

## Introduction

Diabetic nephropathy (DN) is one of the most serious chronic microvascular complications and the key factor that can lead to end-stage nephropathy (Perkovic et al., 2019; Ruiz-Ortega et al., 2020). Although the occurrence and progression of DN can be delayed by the strict control of blood glucose levels and blood pressure levels, these measures cannot completely prevent patients with diabetes from developing into renal failure. Consequently, it is vital to develop new drugs to prevent and treat DN. Renal fibrosis is the main pathological feature of DN (Wei-Jian et al., 2015). In the diabetic state, glomerular mesangial cell proliferation, along with glomerular hypertrophy, gradually lead to the deposition of extracellular matrix (ECM) in the glomerular mesangial region. This leads to the progressive accumulation of inflammatory fibrosis components such as fibronectin (FN) and intercellular cell adhesion molecule-1 (ICAM-1), and can develop into renal fibrosis with glomerulosclerosis as the predominant feature eventually. Oxidative stress is considered to be the main pathogenic factor underlying renal fibrosis in diabetes mellitus (Kultigin, 2017; Sagoo and Gnudi, 2018). In the pathological state of diabetes, high levels of glucose stimulates cells to produce a large number of reactive oxygen species (ROS), which activates a variety of downstream inflammatory signaling pathways, thus inducing and accelerating the occurrence of renal inflammatory fibrosis. Therefore, regulating or inhibiting oxidative stress is an effective method to prevent the progression of diabetic renal fibrosis and thus prevent DN.

The nuclear factor E2-related factor 2 (Nrf2)/antioxidant response element (ARE) signaling pathway is the most important intracellular antioxidant pathway (Hu et al., 2020; Nwakiban et al., 2020). And the activation of Nrf2/ARE pathway can promote the expression of a range of downstream antioxidant enzymes, including heme oxygenase 1 (HO-1) and superoxide dismutase 1 (SOD-1), thereby reducing ROS level and resisting the renal fibrosis injury induced by oxidative stress (Ahmed et al., 2017). However, during end-stage diabetes, the damage caused by the endogenous antioxidant system inevitable leads to the attenuation of Nrf2. Therefore, the activation of Nrf2/ARE signaling pathway exerts a vital protective effect on DN. The family of mammal silent information regulator two proteins (Sirtuins) are deacetylase enzymes that are dependent on nicotinamide adenine dinucleotide (NAD+). Researches have shown that Sirt1 plays a key role in the activation of Nrf2/ARE signaling and the protection of DN (Kim et al., 2018; Arioz et al., 2019; Ma et al., 2019). In addition, studies have shown that Sirt1 could up-regulate the levels of Nrf2 protein and improve oxidative stress (Ma et al., 2018). As such, Sirt1 is considered to be a promising target for the prevention and treatment of DN.

Formononetin (FMN) is the main active ingredient of *Astragalus membranaceus*, Niudali, and other forms of Traditional Chinese medicines with known antioxidant activities (Jin et al., 2017). Formononetin is also known to improve hepatic cholestasis by up-regulating the expression of Sirt1 (Yang et al., 2019). Recent studies have also shown that FMN could improve renal function in diabetic animal models, although the specific mechanisms involved in this effect remain unclear (Qiu et al., 2017). Thus, current evidences indicate that the Sirt1/Nrf2/ARE signaling pathway is important in the prevention and treatment of DN, and that the antioxidant activity of FMN and the agonistic effect of FMN on Sirt1 probably plays a significant role in this process. We hypothesized that the activation of Sirt1/Nrf2/ARE signaling pathway induced by FMN could improve oxidative stress and that FMN probably prevents the progression of diabetic renal fibrosis and thereby represents a useful therapeutic option for patients with DN. In the present study, we used the db/db (diabetic) mouse model and demonstrated that FMN effectively improved the indices of inflammatory fibrosis and oxidative stress. We also found that FMN up-regulated the expression of Sirt1 protein in the renal tissue of db/db diabetic mice and in glomerular mesangial cells (GMCs) under the conditions of high glucose, thus activating the Nrf2/ARE signaling pathway. Our results therefore confirmed that FMN improved diabetic renal fibrosis by activating the of Sirt1/Nrf2/ARE signaling pathway and by relieving oxidative stress.

## Materials and Methods

### Reagents and Antibodies

FMN (purity > 98.0%, HPLC), metformin hydrochloride (Met) (purity > 98.0%, HPLC), and sodium carboxymethyl cellulose (CMC) (purity > 98.0%, HPLC) were provided by Vicci Biotechnology Co., Ltd. (Sichuan, China). Resveratrol (Res) (purity > 99.7%, HPLC) was purchased from MedChemExpress (Monmouth Junction, United States). FN (catalog: 66042-1-1 g, diluted 1:1,000), ICAM-1 (catalog: 16174-1-AP, diluted 1:1,000), Nrf2(catalog: 16,396-1-AP, diluted 1:1,000), Keleh-like ECH-associated protein (Keap1) (catalog: 10503-2-AP, diluted 1:2,500), HO-1 (catalog: 10701-1-AP, diluted 1:2,000) and SOD-1 (catalog: 10269-1-AP, diluted 1:2,000) were purchased from Proteintech Group (Wuhan, China). Sirt1 (catalog: ab110304, diluted 1:1,000) and goat anti-rabbit IgG H&L (Alexa Fluor® 594) (catalog: ab150080)were purchased from Abcam (Cambridge, MA, United States). A superoxide anion fluorescent probe (catalog: S0063), Methylthiazolyldiphenyl-tetrazolium bromide (MTT, catalogue: ST316) and primary anti-diluent, were all provided by Shanghai Biyuntian Biotechnology Co., Ltd. (Shanghai, China). Paraformaldehyde (4%) (catalog: AR1,068) was purchased from Wuhan Baodu Biological Engineering Co., Ltd. (Wuhan, China). RNAiMAX (catalogue: 13778-150) was provided by Seymour Fisher Technologies (Rockford, IL, United States). PAS dye, Masson’s dye, and hematoxylin and eosin (H&E) dye, were provided by Servicebio (Wuhan, China). For the animal work, FMN was dissolved in 0.5% sodium carboxymethyl cellulose. For the *in vitro* work, FMN and Res were both dissolved in Dimethyl sulfoxide (DMSO); Met was dissolved in distilled water.

### Animal Experiments

The mice used in this research were provided by Changzhou Cavins Experimental Animals Co., Ltd. (License number: SCXK 2016–0010). Male db/db mice were divided into five groups: wild db/m (normal control group), db/db (diabetes model group), Met (positive control group; 100 mg/kg), and FMN (FMN treatment group; 25 and 50 mg/kg). Mice in the Met and FMN groups received drugs by oral gavage for eight weeks. Blood and kidney samples were collected from all mice at the end of the eighth week for biochemical and histopathological analyses. All mice were bred at the Laboratory Animal Center of Guangzhou University of Chinese Medicine (License Number: SCXK 2018-0085). The temperature of the animal room was controlled at 23 ± 1°C, relative humidity was controlled at 55 ± 5%, and the photoperiod was controlled at 12 h light/12 h dark. Food and water were available *ad libitum*. All animal experiments are conducted in accordance with relevant regulations relating to experimental animal ethics and were approved by the Animal Experiment Management and Use Committee of Guangzhou University of Traditional Chinese Medicine (Experimental approval reference number: 00222199).

### Biochemical Analyses

Serum levels of blood urea nitrogen (BUN), creatinine (Cr), triglyceride (TG) and total cholesterol (TC) were detected using appropriate detection kits in accordance with the manufacturer’s instructions.

### Cell Culture

The glomeruli from primary GMCs and Sprague-Dawley (SD) rats were isolated and identified by a specificity assay, as described earlier (Xiao et al., 2020). Prior to experiments, GMCs were placed in an incubator set to 37°C and 5% CO_2_. We also supplemented the GMCs with 10% fetal bovine serum (FBS) dulbecco’s modified eagle medium (DMEM) to provide appropriate conditions for growth. The GMCs were then cultured for 2–3 days until they achieved 90% aggregation and were then subcultured at a ratio of 1:6. Cells from the 5th to 14th passages were used for experiments. FMN was dissolved in 100 mM DMSO to provide the stock liquor; this was stored at −20 C. At around 80% confluency, the cells were incubated in sera for 12–16 h and then exposed to hyper-glycemic conditions or FMN for a specified period of time. In this study, the control groups experienced 5.6 mM glucose while the model group experienced 30 mM glucose.

### MTT Assay

In this study, 3-(4, 5-dimethylthiazole-2-yl)-2, 5-diphenyl tetrazolium bromide (MTT, Sigma, United States) was used to evaluate the effect of FMN on the activity of GMCs cells and to determine the appropriate concentration of FMN. Cells were first inoculated in a 96-well plate. Once adherent to the walls of the plate, the cells were cultured in serum-free medium for 12 h and then treated with or without different concentrations of FMN (0, 5, 10, 20, 40, 60, 80, 100 and 150 μM) for 24 h. Next, 20 μM MTT (5 mg/ml) was then added to each well and incubated at 37°C for 4 h before discarding the medium carefully. Finally, DMSO (200 μm) was added to each well to dissolve formazan crystals and a microplate reader (Bio-Tek, United States) was used to measure the absorbance of each well at an optimized wavelength of 570 nm.

### Detection of Intracellular Superoxide

We used a fluorescence probe, dihydroethidium (DHE, Beyotime, Shanghai, China), to detect intracellular levels of ROS. After different treatments (according to experimental requirements), the GMCs cells were washed twice with phosphate buffer solution (PBS); the cells were then incubated with DHE (10 μM) in serum-free DMEM at room temperature for 30 min. Finally, we determined fluorescence intensity by high-content screening (ArrayScan VTI 600 Plus, Thermo Fisher, Rockford, United States).

### Small Interfering RNA and Transient Transfection

Next, we designed a specific small interfering RNA (siRNA) to target Nrf2, along with a negative control; these siRNAs were synthesized by Genepharma (Shanghai, China). The sequences for Sirt1-siRNA were as follows: sense: 5ʹ-CCA​GUA​GCA​CUA​AUU​CCA​ATT-3ʹ, antisense: 5ʹ-UUG​GAA​UUA​GUG​CUA​CUG​GTT-3ʹ. GMCs were inoculated in a 60 mm plate 24 h before the experiment began. Next, we used the RNA iMAX transfection reagent to transfect the cells (at 60% confluency) with appropriate siRNA constructs; the transfection reagent was used in accordance with the manufacturer’s instructions. After 36 h, the medium was removed and 2 ml serum-free DMEM was added to restore cell growth for a further 12 h. Following stimulation, the cells were harvested, and the next experiment was carried out.

### Western Blotting

For western blotting, cultured GMCs or animal tissues were first washed twice with pre-cooled PBS, followed by the addition of RIPA lysis buffer combined with a mixture of proteases or phosphatase inhibitors to help lyse the total protein content of each tissue sample. An equivalent amount of total protein from cells or tissues were then separated by 8–12% sodium dodecyl sulfate-polyacrylamide gel electrophoresis (SDS-PAGE) and then transferred to PVDF membranes (Bio Rad, Hercules, CA, United States). Membranes were first blocked with 5% skimmed milk and then incubated with appropriate primary antibodies at 4 C overnight. The following morning, membranes were washed and then incubated for 1 hat room temperature with the corresponding secondary antibodies. Finally, we used a GE ImageQuant LAS4000mini (GE Healthcare; Waukesha, United States) to observe target bands and then analyzed the relative gray values of different bands with Quantity One protein analysis software (Bio Rad, Hercules, CA, United States).

### Immunofluorescence

GMCs were inoculated on glass cover slips and placed in a 24-well plate in which the cells were exposed to different stimuli. Following treatment, the cells were washed three times in PBS (5 min per wash). The GMCs were then fixed with 4% paraformaldehyde at room temperature for 15 min, dried for 10 min and permeabilized with 1% Triton X-100 dissolved in PBS for 10 min. After washing with PBS, the cells were blocked with goat serum at room temperature for 1 h and then incubated overnight with a rabbit polyclonal antibody raised against Nrf2 (1:200) in a refrigerator at 4 C. The following day, the GMCs were incubated with a secondary antibody for 1 h [Goat anti-rabbit IgG H&L (Alexa Fluor® 594)]. Cell nuclei were labeled with DAPI (5 mg/ml in PBS, Sigma, St. Louis, Mo) for 10 min at room temperature in the dark. Finally, the slides were fixed, and images were acquired using a Zeiss LSM 510 laser confocal fluorescence microscope (Carl Zeiss, Oberkochen, Germany).

### Data Analysis

All data were evaluated by Graphpad Prism 5.0 software (GraphPad Software, Inc., San Diego, CA, United States). Unpaired Student's t-tests were used to compare parameters between two groups. Multiple comparisons were analyzed by one-way analysis of variance (ANOVA) with Bonferroni post hoc tests. *p* < 0.05 was considered to be statistically significant.

## Results

### Formononetin Significantly Improved Indicators of Renal Function in db/db Mice and Reduced Renal Fibrosis

**FIGURE 1:**
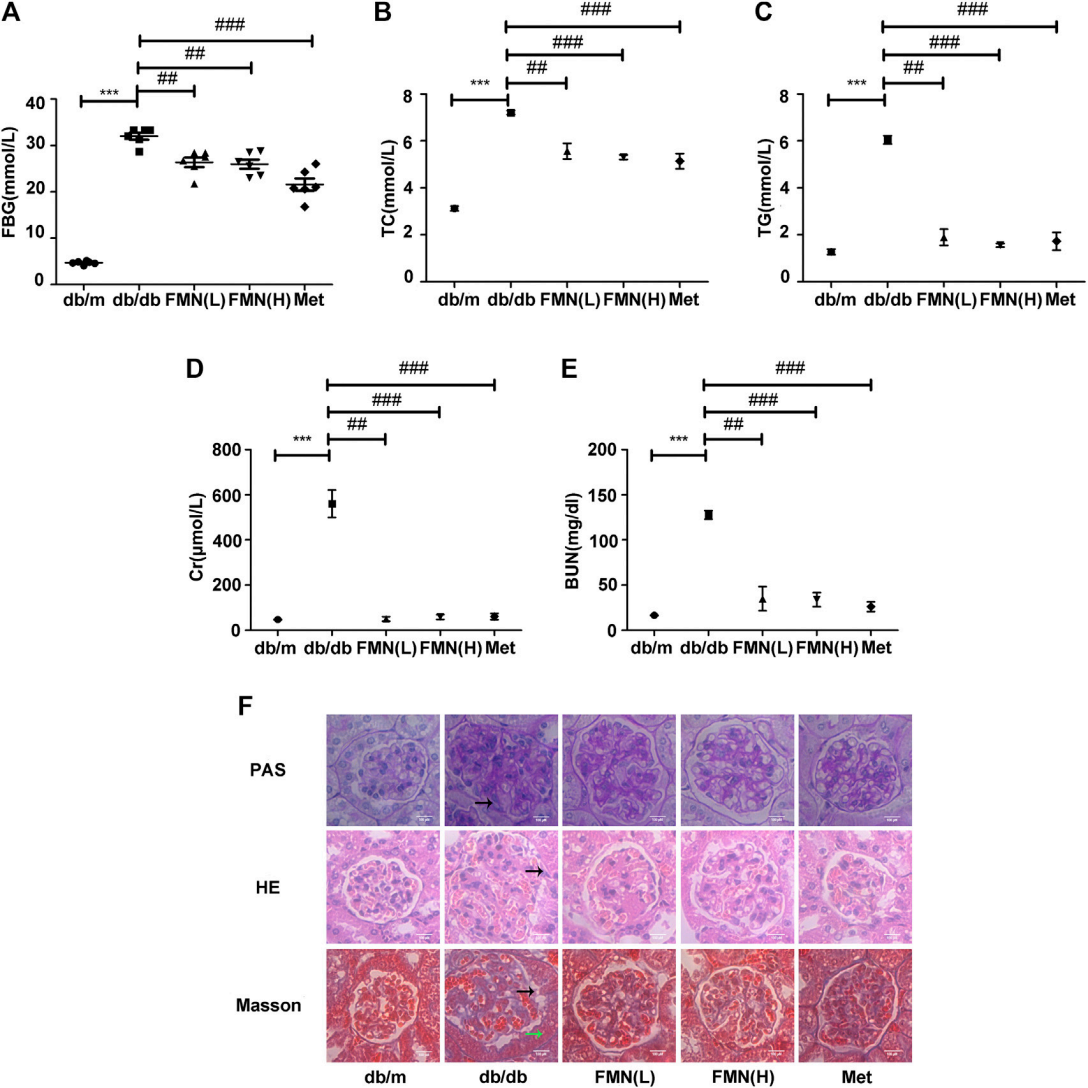
FMN significantly improved indicators of renal function in db/db mice and reduced renal fibrosis. **(A–E)** Levels of FBG, TC, TG, BUN, and Cr, in experimental mice. Data are expressed as ± SD. n = 6, Diabetes: diabetes group; FMN (L): FMN treatment group (low dose: 25 mg/kg); FMN **(H)**: FMN treatment group (medium dose: 50 mg/kg); Met: metformin treatment group (100 mg/kg). ***p < 0.001 vs. db/m, ##*p* < 0.01, ###*p* < 0.001 vs. db/db. **(F)** Histopathological analysis of glomeruli using H&E, PAS, and Masson's staining (400×).

Compared with the control group, the levels of fasting blood glucose (FBG) were significantly higher in the model group (Figure 1A) (*p* < 0.001). However, FMN treatment caused a significant reduction in FBG levels (*p* < 0.01); there was no statistically significant difference between the groups treated with FMN and Met (the positive drug control group). Compared with the model group, FMN significantly reduced the levels of TG, TC, Cr, and BUN (Figures 1B–E) (*p* < 0.01). As shown in Figure 1F, H&E, PAS, and Masson's staining showed that the glomerular structure of mice in the control group remained intact, and no proliferation or glycogen accumulation was observed in the glomerular mesangium. However, in the model group, we observed abnormal hypertrophy of the glomeruli, proliferation of the mesangial cells, an increase in the mesangial matrix, and interstitial fibrosis. Following treatment with FMN, the morphology of the glomeruli gradually returned to normal; the degree of inflammation and fibrosis within the glomeruli also improved (by variable extents). Therefore, these results demonstrated that FMN treatment can significantly improve the pathology of the kidneys in db/db mice.

### Formononetin Increased the Expression of Sirt1 and Nrf2, and Reduced the Expression of Fibronectin and Intercellular Adhesion Molecule 1-1 in the Kidneys of db/db Mice

**FIGURE 2:**
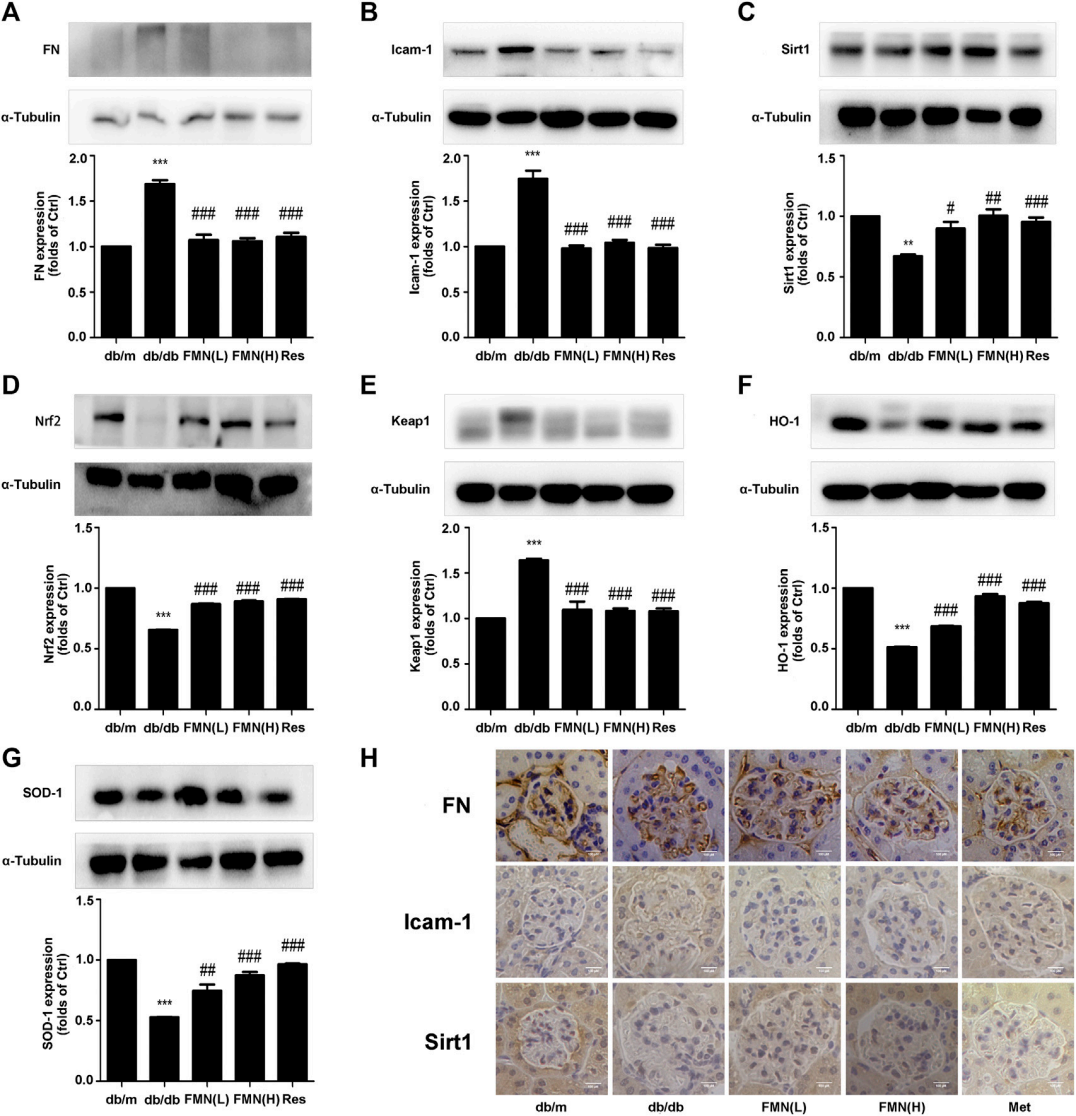
FMN increased the expression of Sirt1 and Nrf2, and reduced the expression of FN and ICAM-1 in the kidneys of db/db mice. **(A–G)** Protein levels of FN, ICAM-1, Sirt1, Nrf2, Keap1, HO-1, and SOD-1, in renal tissues from diabetic mice, as detected by western blotting. **p < 0.01, ***p < 0.001 vs. db/m, #*p* < 0.05, ##*p* < 0.01, ###*p* < 0.001 vs. db/db. **(H)** Immunohistochemical staining of FN, Icam-1, and Sirt1 in glomeruli (400×). Independent experiments were performed at least three times (with similar results).

In order to further explore the potential mechanisms that might underlie the ability of FMN to resist oxidative stress and alleviate diabetic kidney fibrosis, we tested a range of associated proteins. As shown in Figures 2A–G, the protein levels of FN, ICAM-1, and Keap1, decreased significantly in mice treated with FMN (*p* < 0.001), while the protein levels of Nrf2, HO-1, SOD-1, and Sirt1 increased (*p* < 0.05). Immunohistochemical results (Figure 2H) also confirmed that FMN treatment improved Sirt1 expression and inhibited the high expression levels of FN and ICAM-1 in db/db mice. These results suggest that the mechanism by which FMN improves renal fibrosis in diabetic mice may be related to the up-regulation of Sirt1 and the activation of the Nrf2/ARE signaling pathway. To further confirm this hypothesis, we next conducted a series of *in vitro* experiments.

### Formononetin Inhibited Fibronectin and Intercellular Adhesion Molecule-1 Protein Levels and Reduced Levels of Reactive Oxygen Species in Glomerular Mesangial Cells Induced by High Glucose

**FIGURE 3:**
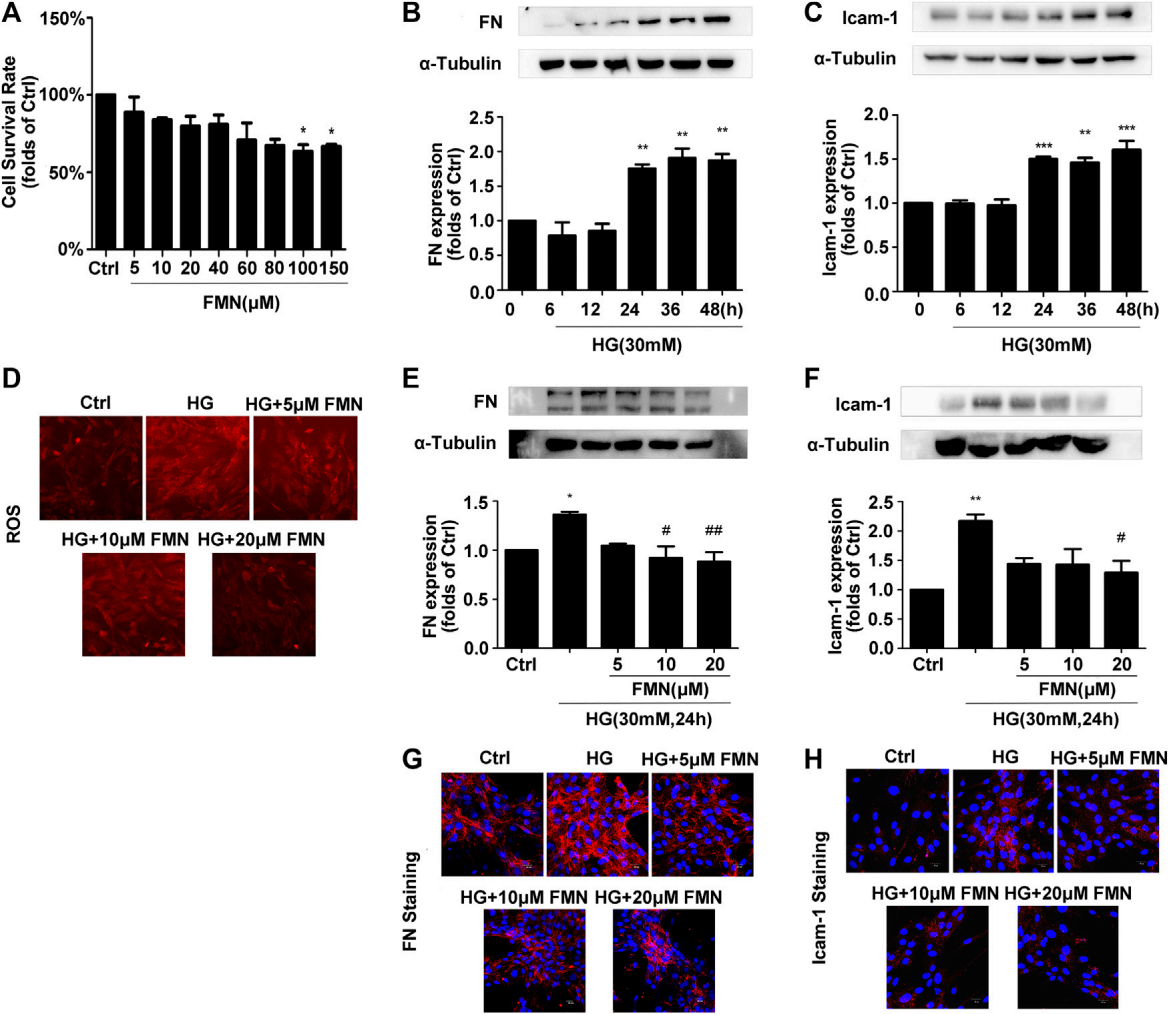
FMN inhibited FN and ICAM-1 protein levels and reduced levels of ROS in GMCs induced by high glucose. **(A)** The viability of GMCs, as detected by MTT assay, *p < 0.05 vs. control. **(B,C)** The expression levels of FN and ICAM-1 in GMCs, as detected by western blotting, **p < 0.01, ***p < 0.001 vs. 0 h. **(D)** FMN inhibited the high ROS levels induced by high glucose conditions. **(E,F)** The protein levels of FN and ICAM-1 in GMCs, as determined by western blotting, *p < 0.05, **p < 0.01 vs. Ctrl, #*p* < 0.05, ##*p* < 0.01 vs. HG. **(G,H)** The distribution of FN and ICAM-1, as determined by immunofluorescence (magnification 400×). Red fluorescence indicates the localization of FN and ICAM-1. Independent experiments are performed at least three times with similar results.

MTT data showed that when applied at a concentration of 100 μM, FMN had no cytotoxic effects on GMCs (Figure 3A) (*p* < 0.05). The results of a preliminary dose test showed that FMN could inhibit the expression of FN and ICAM-1 in GMCs stimulated by high glucose at a concentration of 5 μM (Figures 3E,F) (*p* < 0.05). Therefore, 5, 10 and 20 μM of FMN were used in subsequent studies. The expression levels of FN and ICAM-1 in GMCs increased in a time-dependent manner following stimulation with high glucose at specified times (0, 6, 12, 24, 36, and 48 h) (*p* < 0.01). Compared with the control group, the upregulation of these indicators was triggered by 30 mM hyper-glycemia for 24 h (Figures 3B,C) (*p* < 0.05), as shown by western blotting. Therefore, stimulation with high glucose levels (30 mM) for 24 h was used as a modelling condition for all subsequent experiments. In addition, western blotting and immunofluorescence experiments further showed that FMN treatment reduced the up-regulated expression of FN and ICAM-1 induced by high glucose stimulation for 24 h in a dose-dependent manner (Figures 3E–H) (*p* < 0.05). Furthermore, the levels of ROS in GMCs stimulated by high glucose conditions were significantly higher than those in the control group, while levels of ROS in the FMN-treated group were significantly reduced (Figure 3D).

### Formononetin Increased the Nuclear Translocation of Nrf2 to Activate the Nrf2/ARE Signaling Pathway

**FIGURE 4:**
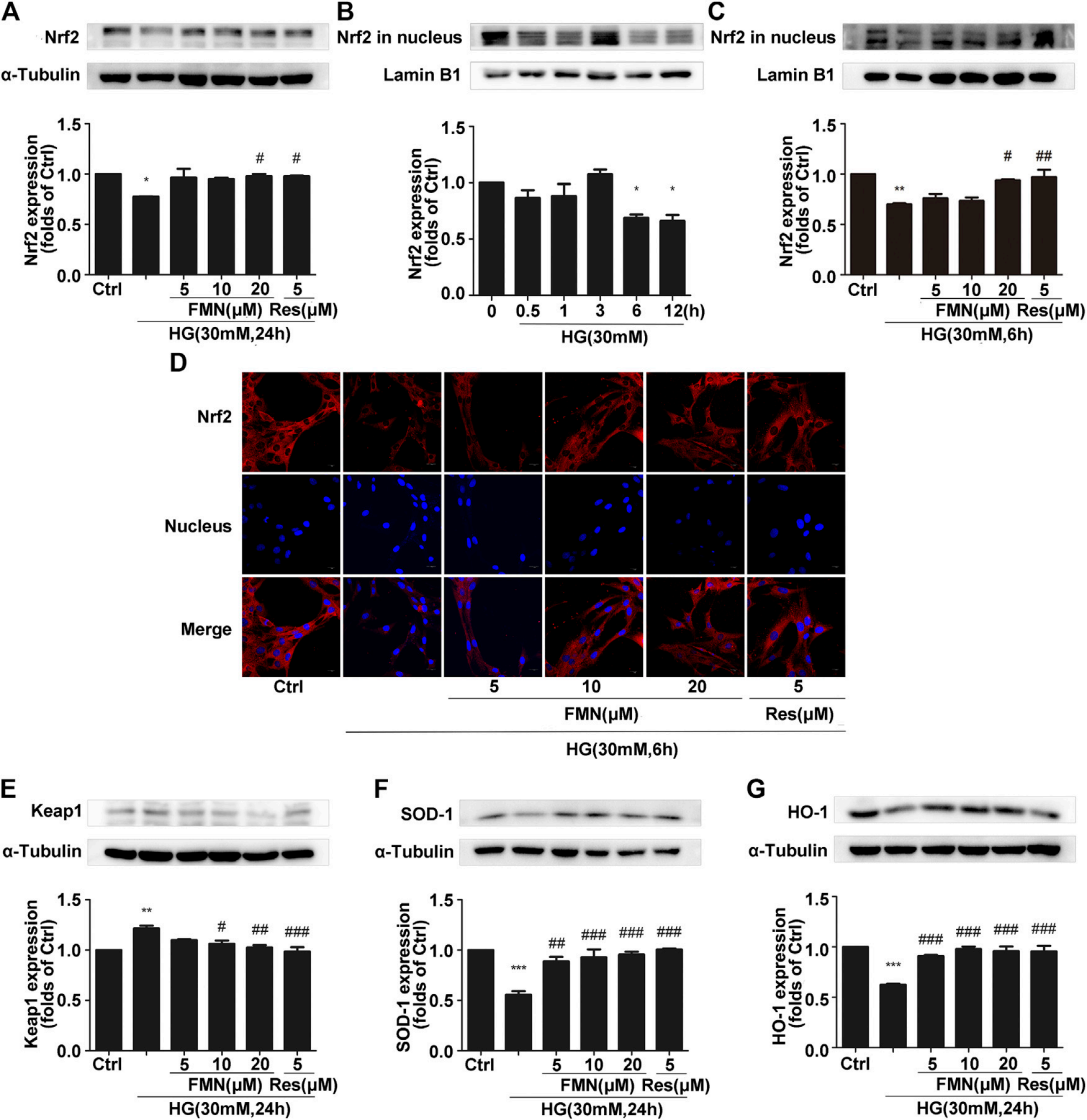
FMN increased the nuclear translocation of Nrf2 to activate the Nrf2/ARE signaling pathway. **(A)** The protein levels of Nrf2 in GMCs, as determined by western blotting, *p < 0.05 vs. control, #*p* < 0.05 vs. HG. **(B)** The expression of Nrf2 in GMCs, as determined by western blotting. *p < 0.05 vs. 0 h. **(C)** The protein levels of Nrf2 in GMCs, as determined by western blotting, **p < 0.01 vs. control (Ctrl), #*p* < 0.05, ##*p* < 0.01 vs. HG. **(D)** The distribution of Nrf2 in GMCs, as determined by immunofluorescence analysis. Scale bar: 20 μm. Red fluorescence indicates the localization of Nrf2. **(E–G)** The protein levels of Keap1, SOD-1, and HO-1, in GMCs, as determined by western blotting, **p < 0.01, ***p < 0.001 vs. control, ##*p* < 0.01, ###*p* < 0.01 vs. HG. Independent experiments were performed at least three times (with similar results).

In order to verify the correlation between the activation of the Nrf2/ARE signaling pathway by FMN and the enhancement of Sirt1 expression by FMN, we used resveratrol, a classic agonist of Sirt1, as a positive control. As shown in Figure 4A, the total Nrf2 protein content in GMCs from the FMN treatment group was significantly increased compared with that of the control group (*p* < 0.05). In order to explore the activation effect of FMN on the nuclear expression of Nrf2, we conducted aging experiments on Nrf2 nuclear protein; data showed that 30 mM high glucose stimulation for 6 h represented the optimal conditions for subsequent experiments (Figure 4B) (*p* < 0.05). Western blotting showed that FMN treatment increased the nuclear expression of Nrf2 (*p* < 0.05). Immunofluorescence experiments also showed that Nrf2 was distributed inside and outside of the signaling nucleus in GMCs under physiological conditions, but levels of Nrf2 were reduced in the nucleus under high glucose stimulation. Following FMN treatment, the expression of Nrf2 protein in the nucleus increased (Figures 4C,D) (*p* < 0.05). The expression of the Nrf2 target genes, HO-1 and SOD-1, were also increased following the administration of FMN (Figures 4F,G) (*p* < 0.001). Keap1 has the ability to regulate the stability of Nrf2 protein and inhibit its expression, so the inhibitory effect of formononetin on Keap1 is also worthy of attention (Vittorio et al., 2010). Meanwhile, in this experiment, the increased expression of Keap1 induced by high glucose stimulation was further reduced in the FMN-treated group (Figure 4E) (*p* < 0.05).

### Formononetin Increased the Expression of Sirt1 in Glomerular Mesangial Cells Induced by High Glucose

**FIGURE 5:**
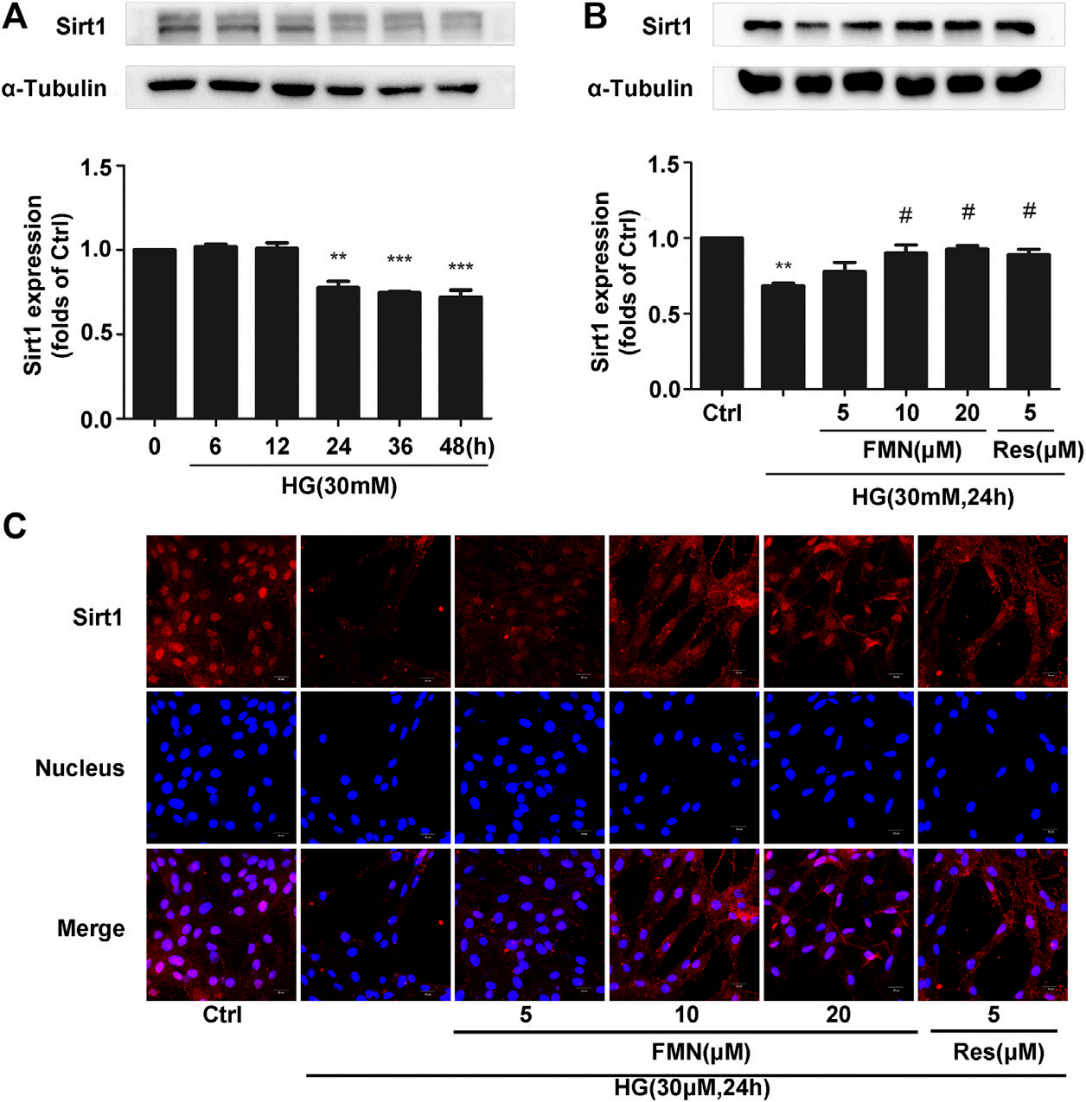
FMN increased the expression of Sirt1 in GMCs induced by high glucose. **(A)** The protein levels of Sirt1 in GMCs, as determined by western blotting, **p < 0.01, ***p < 0.001 vs. 0 h. **(B)** The protein levels of Sirt1 in GMCs, as determined by western blotting, **p < 0.01 vs. control, #*p* < 0.05 vs. HG. **(C)** Distribution of Sirt1 in GMCs, as determined by immunofluorescence. Scale bar: 20 μm. Red fluorescence indicates the localization of Sirt1. Independent experiments were performed at least three times (with similar results).

Collectively, the previous experiments demonstrated that FMN was clearly able to activate the Nrf2/ARE signaling pathway. Furthermore, our animal experiments showed that FMN was also activated the expression of Sirt1. In order to verify this more accurately, we conducted aging experiments and finally determined the appropriate conditions to use in subsequent experiments; we ascertained that we should apply 30 mM high-glucose stimulation for a period of 24 h (Figure 5A) (*p* < 0.01). As shown in Figure 5B, FMN alleviated the inhibitory effect of Sirt1 caused by high-glucose stimulation (*p* < 0.05); this was consistent with data derived from animal experiments. This was further supported by our immunofluorescence data (Figure 5C).

### The Depletion of Sirt1 Inhibited the Activation of the Nrf2/ARE Signaling Pathway Induced by Formononetin

**FIGURE 6:**
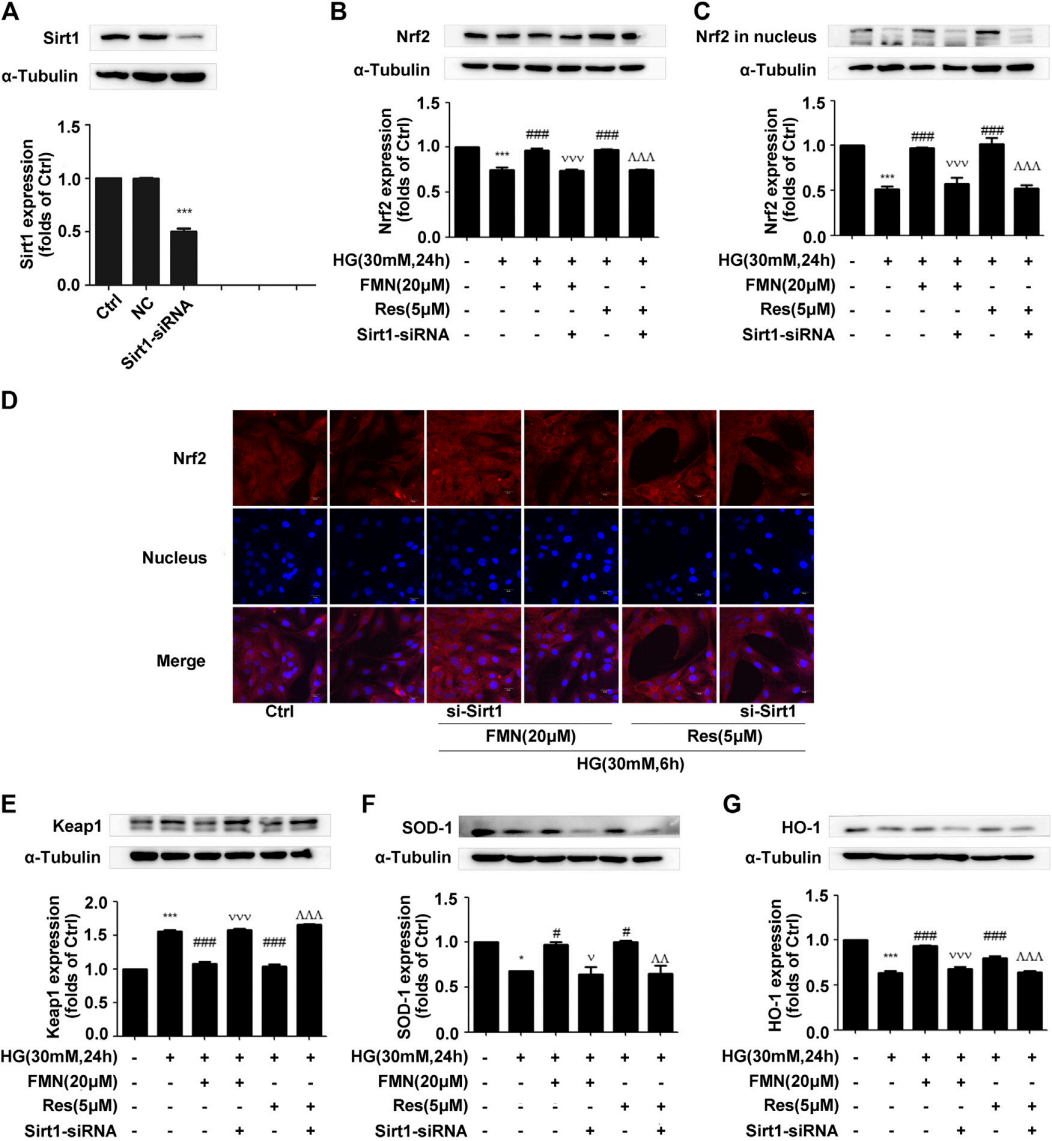
The depletion of Sirt1 inhibited the activation of the Nrf2/ARE signaling pathway induced by FMN. **(A)** The protein levels of Sirt1 in GMCs, as determined by western blotting, ***p < 0.001 vs. control (Ctrl). **(B,C)** The protein levels of Nrf2 in GMCs, as determined by western blotting, ***p < 0.001 vs. control, ###*p* < 0.001 vs. HG, vvv *p* < 0.001 vs. HG with FMN, ^  ^  ^*p* < 0.001 vs. HG with Res. **(D)** The distribution of Nrf2 in GMCs, as determined by immunofluorescence. Scale bar: 20 μm. Red fluorescence indicates the localization of Nrf2. **(E–G)** The protein levels of Nrf2 in GMCs, as determined by western blotting, *p < 0.05, ***p < 0.001 vs. control (Ctrl), #*p* < 0.05, ###*p* < 0.001 vs. HG, v *p* < 0.05, vvv *p* < 0.001 vs. HG with FMN, ^  ^*p* < 0.01, ^  ^  ^*p* < 0.001 vs. HG with Res. Independent experiments were performed at least three times (with similar results).

According to the previous experiments, we ascertained that FMN plays an important role in activating the Nrf2/ARE signaling pathway and improving the process of diabetic renal fibrosis. In order to further clarify the significance of Sirt1 in this process, we used siRNA constructs to interfere with the expression of Sirt1 (Figure 6A) (*p* < 0.001). Data showed that the total Nrf2 protein content was significantly reduced when FMN and Sirt1-siRNA were administered at the same time (Figure 6B) (*p* < 0.001). Immunofluorescence and western blotting data also showed that the distribution of Nrf2 protein in the nucleus also decreased following the administration of FMN and Sirt1-siRNA (Figures 6C,D) (*p* < 0.001); the expression of Keap1 increased, and the levels of the Nrf2 target proteins, HO-1 and SOD-1, were also reduced to different degrees (Figures 6E–G) (*p* < 0.05).

### The Depletion of Sirt1 Reversed the Effects of Formononetin on the Inhibition of Oxidative Stress

**FIGURE 7:**
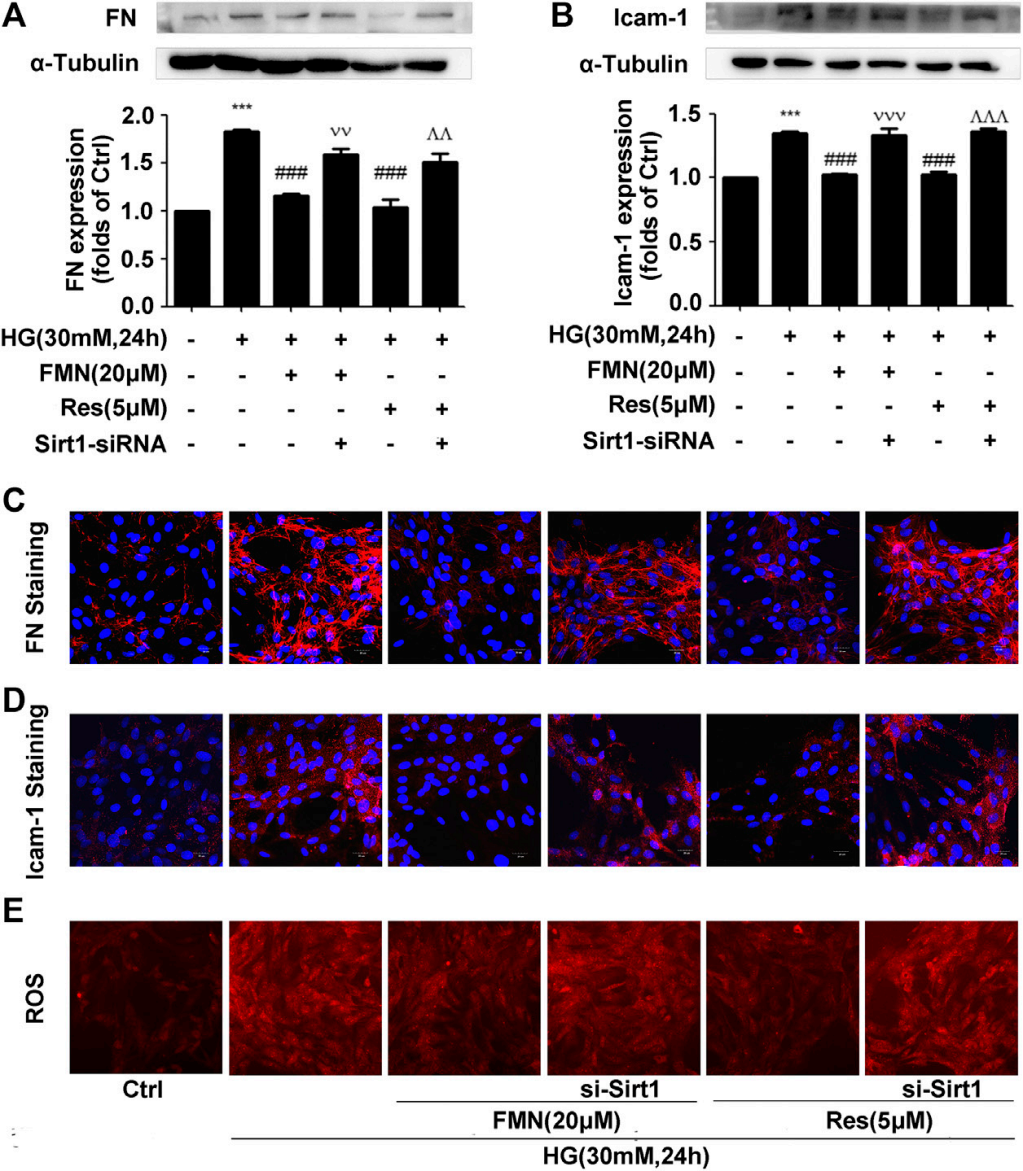
The depletion of Sirt1 reversed the effects of FMN on the inhibition of oxidative stress. **(A,B)** The protein levels of FN and ICAM-1 in GMCs, as determined by western blotting, ***p < 0.001 vs. control (Ctrl), ###*p* < 0.001 vs. HG, *p* < 0.001 vs. HG with FMN, ^  ^  ^*p* < 0.001 vs. HG with Res. **(C,D)** The distribution of FN and ICAM-1 in GMCs, as determined by immunofluorescence. Scale bar: 20 μm. Red fluorescence indicates the localization of FN and ICAM-1. **(E)** The depletion of Sirt1 further enhanced ROS levels induced by HG. Independent experiments were performed at least three times (with similar results).

Activation of the Nrf2/ARE pathway by FMN was inhibited following interference of the Sirt1 protein. In order to verify whether activation of the Nrf2/ARE pathway was also inhibited, we investigated the protein levels of FN and ICAM-1. Western blotting and immunofluorescence experiments showed that Sirt1 interference alleviated the reduced expression levels of FN and ICAM-1 induced by FMN (Figures 7A–D) (*p* < 0.001). Sirt1 interference also prevented FMN from lowering the intracellular levels of ROS (Figure 7E).

## Discussion

Studies have shown that isoflavones could improve the oxidative stress effect by inhibiting the heat shock signaling pathway (Vittorio et al., 2010). FMN as an isoflavone compound also has been widely reported to improve the renal function of patients suffering from diabetes, and enhance resistance to oxidative stress (Kim et al., 2018; Zhen et al., 2019). In addition, several previous studies have reported that FMN could exert a range of pharmacological activities, including anti-atherosclerosis, anti-tumor, and selective neuroprotective effects (El-Bakoush and Olajide, 2018; Ong et al., 2019; Ma et al., 2020). Oxidative stress is a key link in the intensification of renal fibrosis and can also accelerate the progression of diabetic nephropathy. Given that FMN has been reported to exert significant antioxidant capacity, we hypothesized that FMN could also improve diabetic nephropathy via its antioxidant capabilities probably.

In this study, we used db/db mice as a model for type 2 diabetic nephropathy as experimental animals. We found that FMN significantly reduced the levels of BUN, Cr, TG, and TC in the model group, thus indicating that FMN could clearly improve the renal function of diabetic mice. Furthermore, HE, PAS, and Masson’s staining showed that after FMN treatment, there was clear improvements in renal tubular interstitial fibrous tissue hyperplasia and glycogen accumulation compared with the model group. Endogenous cellular defense mechanism plays an important role in resisting oxidative stress and maintaining cellular state. The Nrf2/ARE signaling pathway is one of the most well-studied oxidative stress pathways. Studies have shown that Nrf2 could maintain protein homeostasis under various pathological conditions and improve oxidative stress by encoding the antioxidant pathway of Vitagenes (Calabrese et al., 1996; Calabrese et al., 2010; Calabrese and Calabrese, 2013). It is generally believed that when Nrf2 is stimulated by oxidation, it is separated from endogenous inhibitor (Keap1) and then Nrf2 translocates into the nucleus to mediate the transcription of its target genes, including HO-1 and SOD-1 (Gong et al., 2018). In order to study the antioxidant effects of FMN, we carried out western blotting and immunofluorescence experiments using both mouse kidney tissue and GMCs that had been induced by high glucose. Data showed that FMN treatment could activate the Nrf2/ARE signaling pathway and significantly enhance the nuclear translocation of Nrf2. FMN also alleviated renal fibrosis by reducing the expression levels of FN and ICAM1both *in vitro* and *in vivo*. Some studies have shown that Sirt1 is closely associated with the pathogenesis of diabetic nephropathy and represents a new potential therapeutic target for diabetic nephropathy (Munehiro et al., 2013; Sun et al., 2018). Sirt1 agonists are known to have protective effects on the metabolism of some diabetic animals, including glucose tolerance, fasting glucose level, and insulin resistance (Marina et al., 2018). Sirt1 knockout mice showed increased levels of proteinuria and renal impairment (Hasegawa et al., 2013). Furthermore*, in vitro* and *in vivo* studies have shown that Res, an agonist of Sirt1, could reduce ROS production and up-regulate the expression of endogenous antioxidant genes and vascular protective molecules (Vella et al., 2015; Yang et al., 2018). In this study, we found that FMN promotes the expression of Sirt1 both *in vitro* and *in vivo*. In addition, we knocked out Sirt1 in GMCs induced by high glucose, and found that the antioxidant and anti-fibrosis effects induced by FMN were negated. Although we have demonstrated that FMN can prevent the deposition of ECM and improve oxidative stress by regulating the Sirt1/Nrf2/ARE pathway, how does FMN directly activate Sirt1 and its association with Vitagenes network have not been fully clarified. This issue needs further investigation. Collectively, these results suggest that Sirt1 is necessary for FMN to activate the Nrf2/ARE signaling pathway.

## Conclusion

*In vitro* and *in vivo* experiments confirmed that the inhibitory mechanism underlying the effects of FMN on diabetic renal fibrosis was closely related to the up-regulation of Sirt1 expression and activation of the Nrf2/ARE signaling pathway. Our results provide an experimental basis for the application of FMN in the prevention and treatment of DN and the development of new drugs.

## Data Availability Statement

The raw data supporting the conclusions of this article will be made available by the authors, without undue reservation.

## Ethics Statement

The animal study was reviewed and approved by Animal Ethics Committee of Guangzhou University of Chinese Medicine. Written informed consent was obtained from the owners for the participation of their animals in this study.

## Author Contributions

KZ and HH designed and performed the experiments, acquired and analyzed the data, and drafted the manuscript. XJ and CY helped to perform the animal experiments. YW, RL, and YMW helped to draft the manuscript. SQ contributed reagents, materials, and analysis tools. All authors read and approved the final manuscript.

## Funding

This research was supported by the 2019 Guangdong University Characteristic Innovation Project (Reference number: 2019KTSCX023).

## Conflict of Interest

The authors declare that the research was conducted in the absence of any commercial or financial relationships that could be construed as a potential conflict of interest.
