# Supplementary material for: Formononetin Activates the Nrf2/ARE Signaling Pathway Via Sirt1 to Improve Diabetic Renal Fibrosis
Source: Front Pharmacol. 2021 Jan 13;11:616378. doi: 10.3389/fphar.2020.616378 (PMC7845558; doi:10.3389/fphar.2020.616378)
Supplement: Supplementary file 1 [file datasheet1.docx]

Supplementary Material

**Supplementary Figure1**


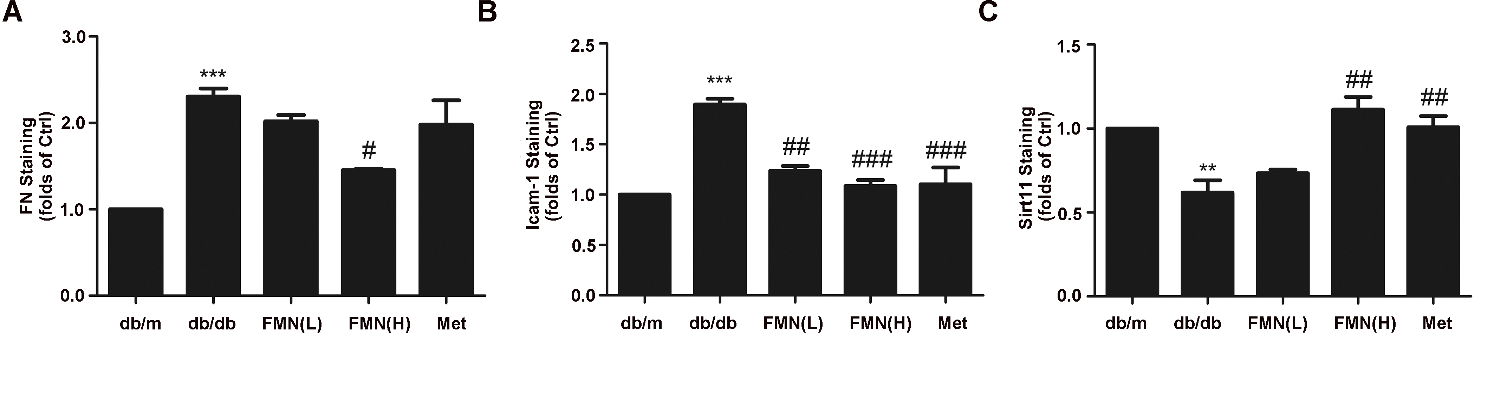


Figure S1. FMN increased the expression of Sirt1 and Nrf2 , and reduced the expression of FN and ICAM-1 in the kidneys of db/db mice.(A-C) Statistical analysis of FN,Icam1 and Sirt1 protein level. **P<0.01, ***P<0.001 *vs*. db/m, #P<0.05, ^##^P<0.01, ^###^P<0.001 *vs.* db/db.Independent experiments were performed at least three times (with similar results).

**Supplementary Figure 2**


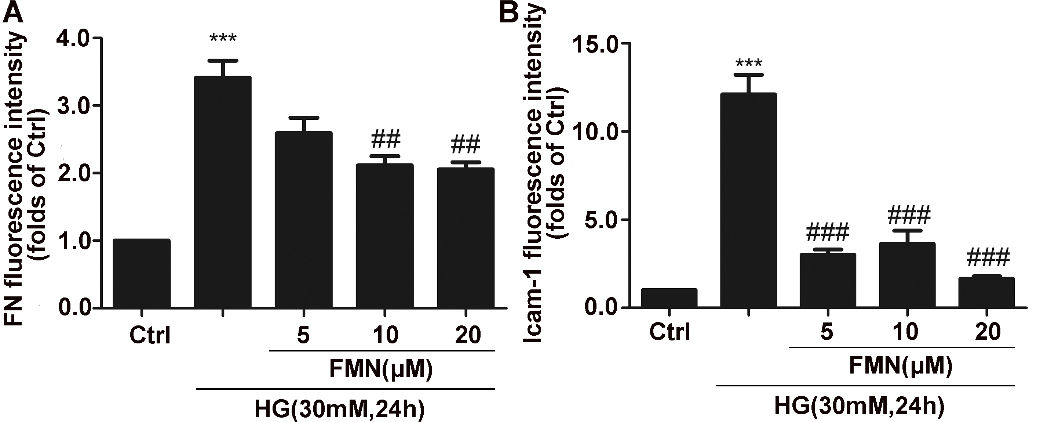


Figure S2. FMN inhibited FN and ICAM-1 protein levels and reduced levels of ROS in GMCs induced by high glucose. (A-B) The fluorescence intensity of FN and Icam1,***P<0.001 *vs*. Ctrl, ^##^P<0.01, ^###^ P<0.001 *vs*. HG. Independent experiments are performed at least three times with similar results.

**Supplementary Figure 3**


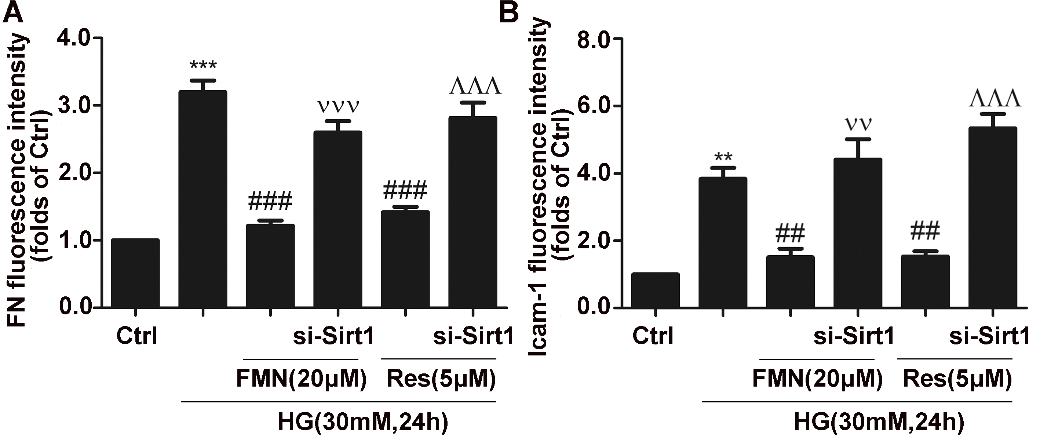


Figure S3. The depletion of Sirt1 reversed the effects of FMN on the inhibition of oxidative stress. (A-B) The fluorescence intensity of FN and Icam1,^**^ P < 0.01, ^***^ P< 0.001 *vs*. control (Ctrl),^##^ P< 0.01, ^###^P< 0.001 *vs.* HG, ^ⅴⅴ^ P< 0.01, ^ⅴⅴⅴ^ P< 0.001 *vs.* HG with FMN, ^^^P< 0.001 *vs.* HG with Res. Independent experiments were performed at least three times (with similar results).
